# A rare mechanism of embolic stroke complicating coronary thrombus aspiration

**DOI:** 10.1002/ccr3.6951

**Published:** 2023-02-21

**Authors:** Reiko Shiomura, Hideki Miyachi, Takeshi Yamamoto, Hitoshi Takano

**Affiliations:** ^1^ Division of Cardiovascular Intensive Care Nippon Medical School Hospital Tokyo Japan; ^2^ Department of Cardiovascular Medicine Nippon Medical School Tokyo Japan

**Keywords:** contrast agent, myocardial infarction, stroke, thrombus, thrombus aspiration

## Abstract

Aspiration thrombectomy is often performed in patients with acute myocardial infarction with high thrombus burden. Current guidelines, however, recommend against it because of stroke risk. We report a case of embolic stroke complicating coronary thrombus aspiration in a 62‐year‐old man. Aspiration thrombectomy during percutaneous coronary intervention migrated thrombus to the proximal right coronary artery (RCA), and the thrombus was subsequently released into the aorta by backflow of the contrast injection causing aspiration thrombectomy‐associated stroke. This is an extremely rare mechanism by which complications arise from failed aspiration thrombectomy.

## 1 INTRODUCTION

Current guidelines do not recommend routine aspiration thrombectomy for patients with acute myocardial infarction (AMI), however, it is still one of the standard treatments during percutaneous coronary intervention (PCI) in AMI patients with high thrombus burden. Aspiration thrombectomy has a risk of stroke, and the mechanism involves incompletely aspirated thrombus dislodge inside the guide catheter and subsequently be injected into the systemic circulation.

Here, we experienced a case of embolic stroke complicating coronary thrombus aspiration due to an extremely rare mechanism different from that described above. We share the rare case to avoid similar complications in the future.

## 2 CASE REPORT

A 62‐year‐old man presented to his family doctor with a history of chest pain at rest for the past 4 days. He was diagnosed with non‐ST segment elevation myocardial infarction (NSTEMI) and was referred to our institute. His blood pressure and heart rate were 134/67 mmHg and 81 beats/min, respectively. The physical examination revealed a high body mass index (27.6 kg/m^2^) and normal heart sounds without peripheral edema.

**FIGURE 1:**
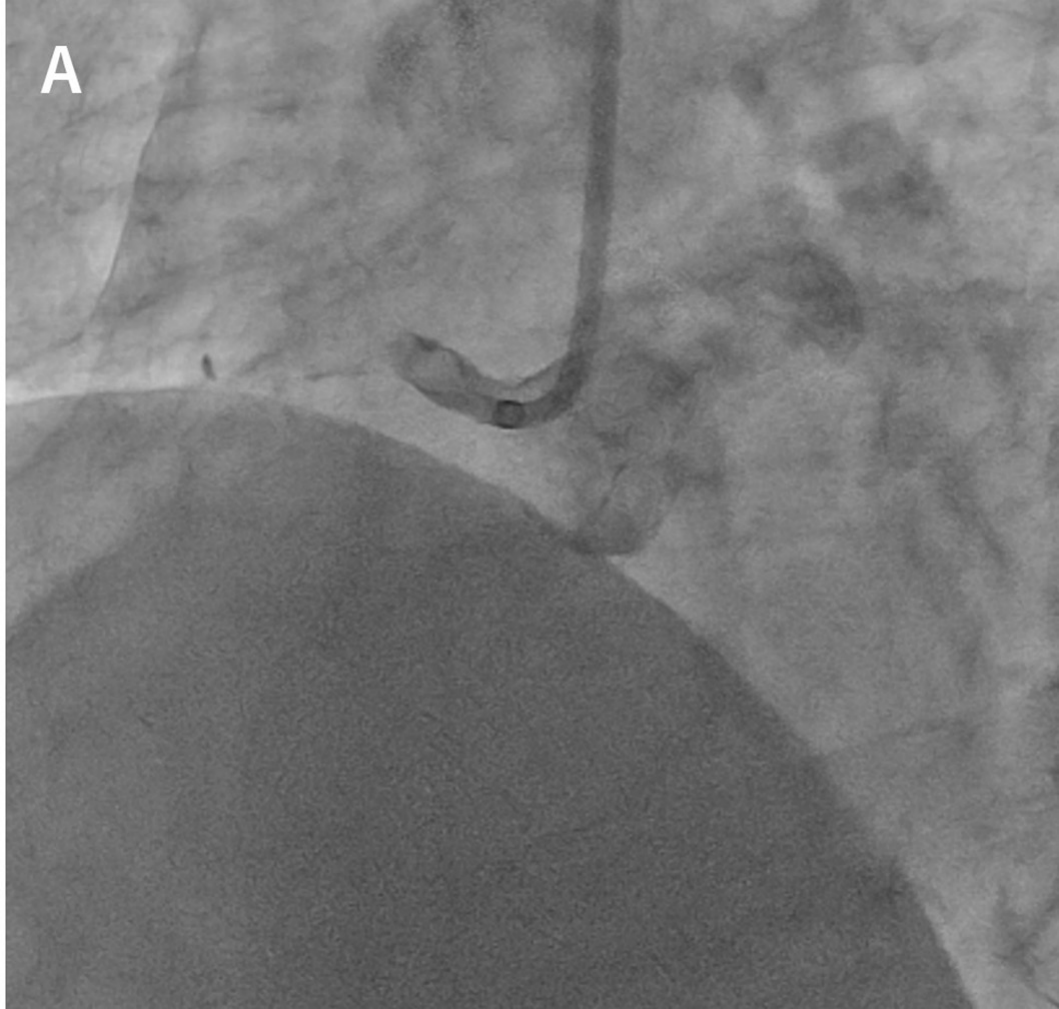
Initial coronary angiography. Coronary angiography shows total thrombotic occlusion at the ostial right coronary.

**FIGURE 2:**
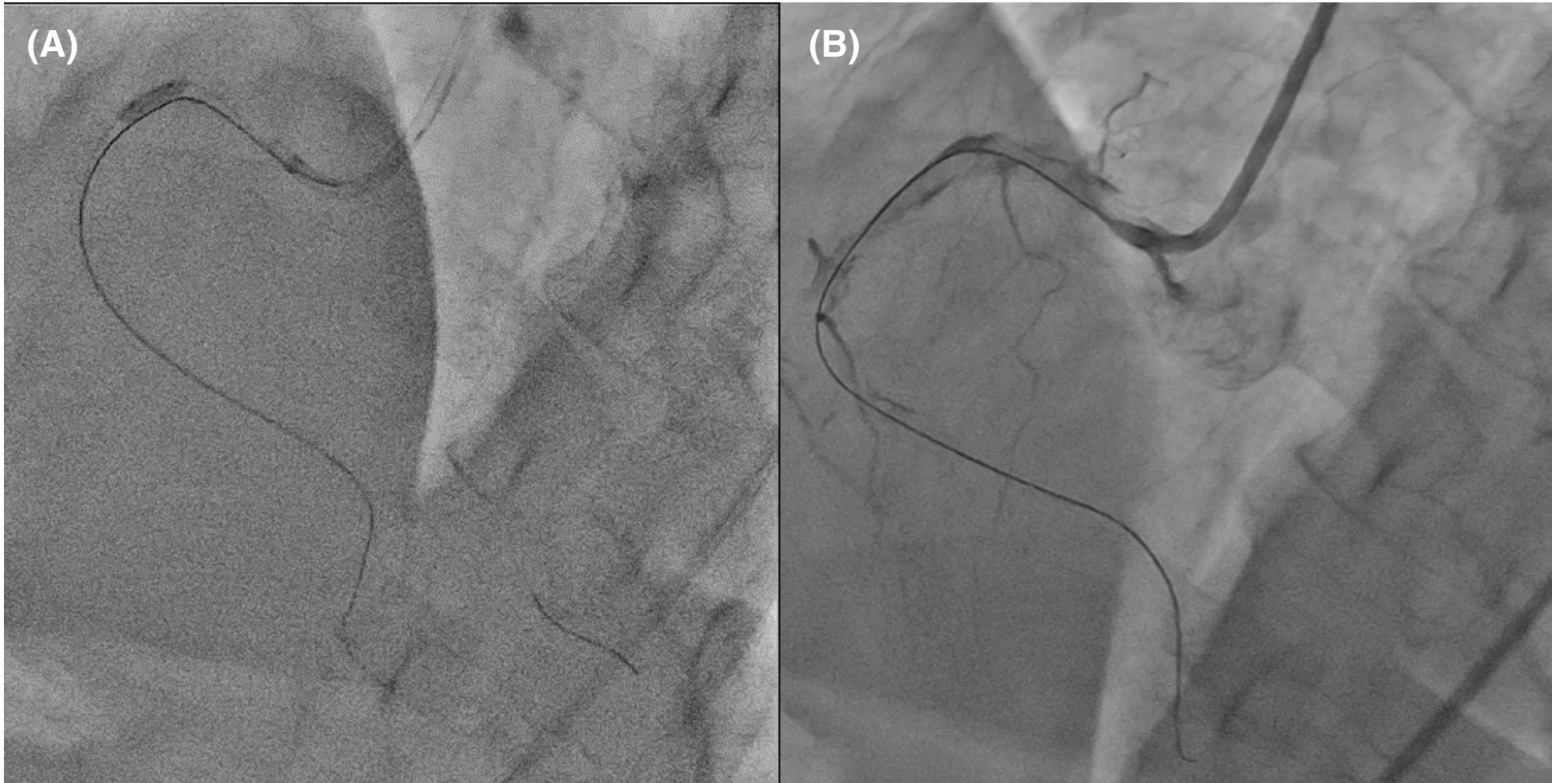
Balloon dilatation (A) and right coronary angiography after balloon dilatation (B). Balloon dilatation is performed on the proximal lesions (A). A high thrombus burden remains in the right coronary artery (B).

**FIGURE 3:**
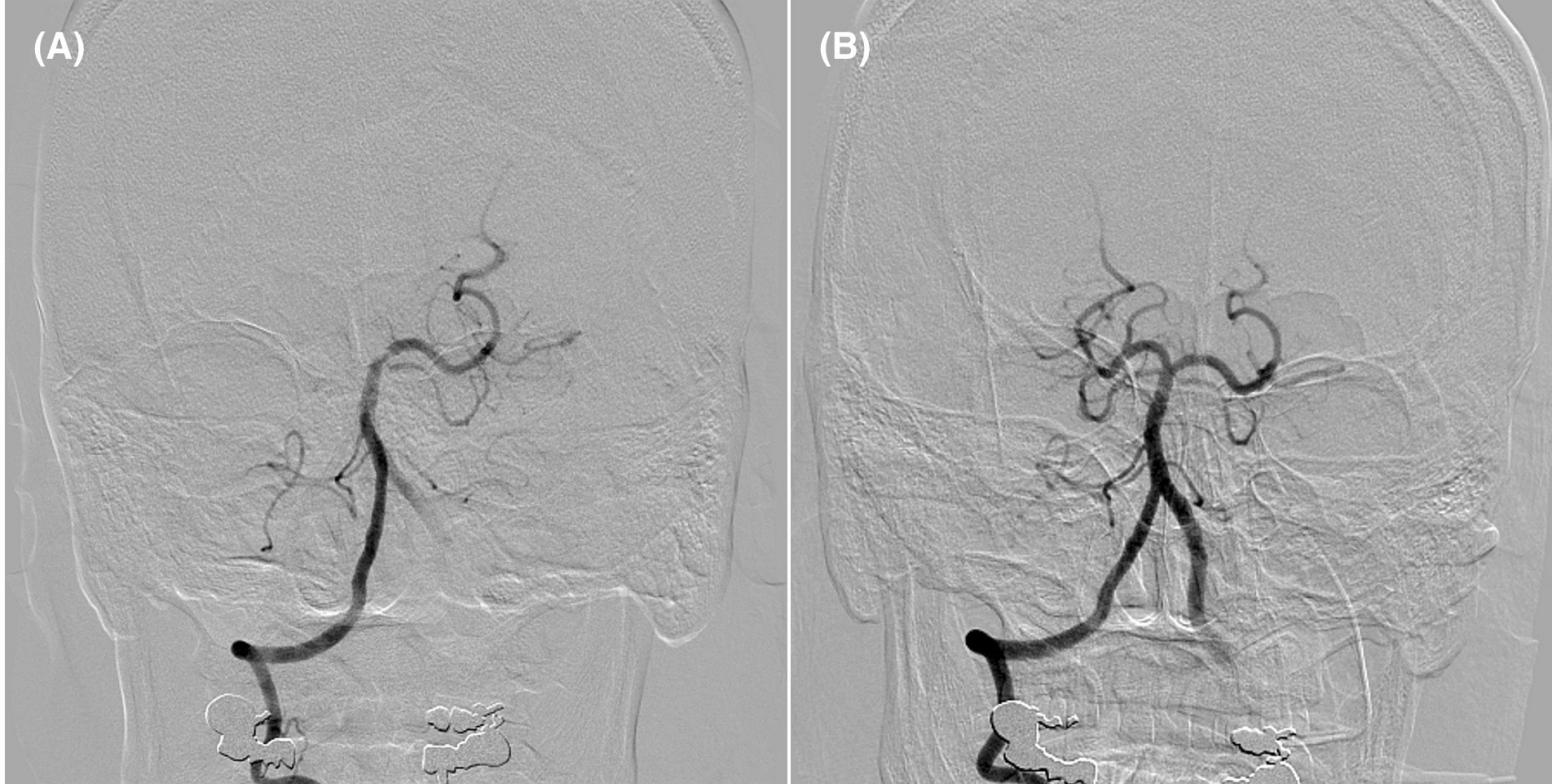
Cerebral angiography. Cerebral angiography showing complete occlusion of the right posterior cerebral artery (A). The right posterior cerebral artery is reperfused after endovascular thrombectomy (B).

**FIGURE 4:**
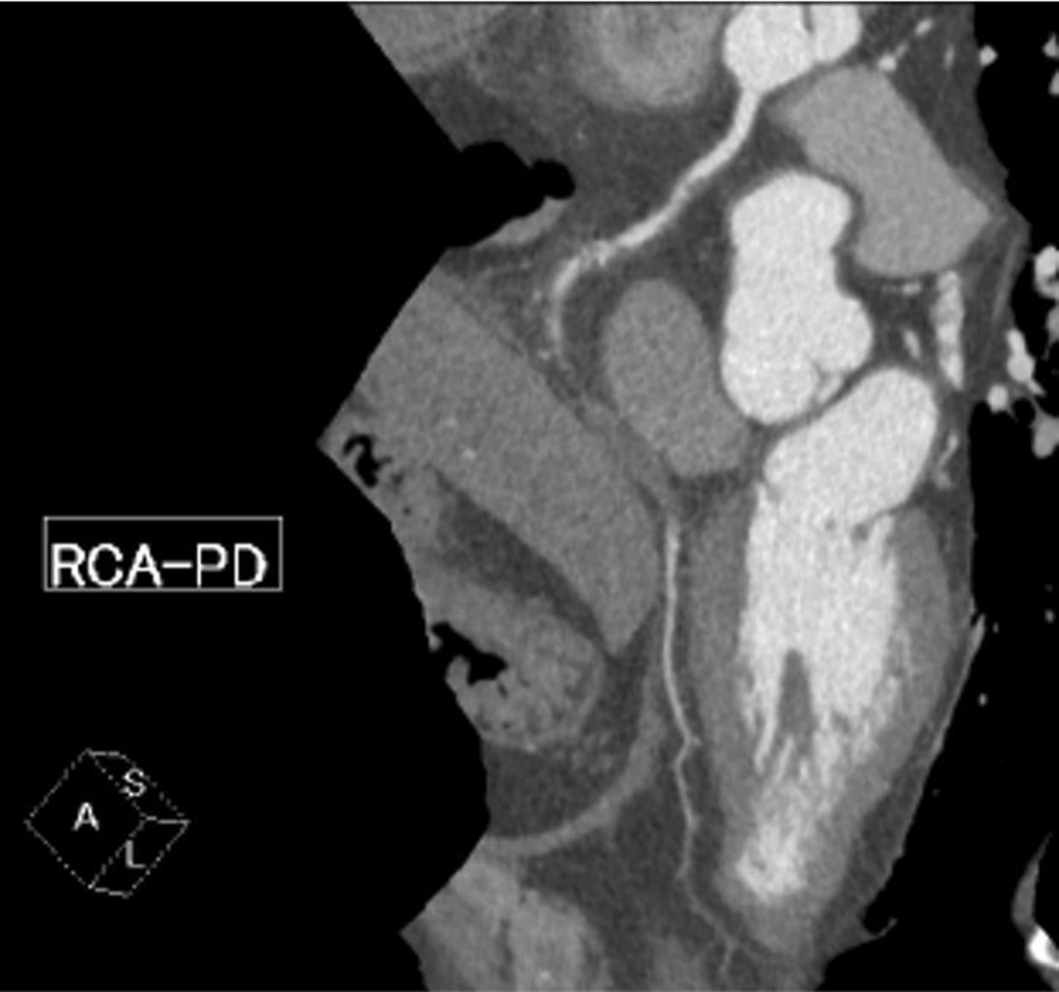
Coronary computed tomography angiography. Follow‐up coronary computed tomographic angiography reveals a residual thrombus in the right coronary artery.

**FIGURE 5:**
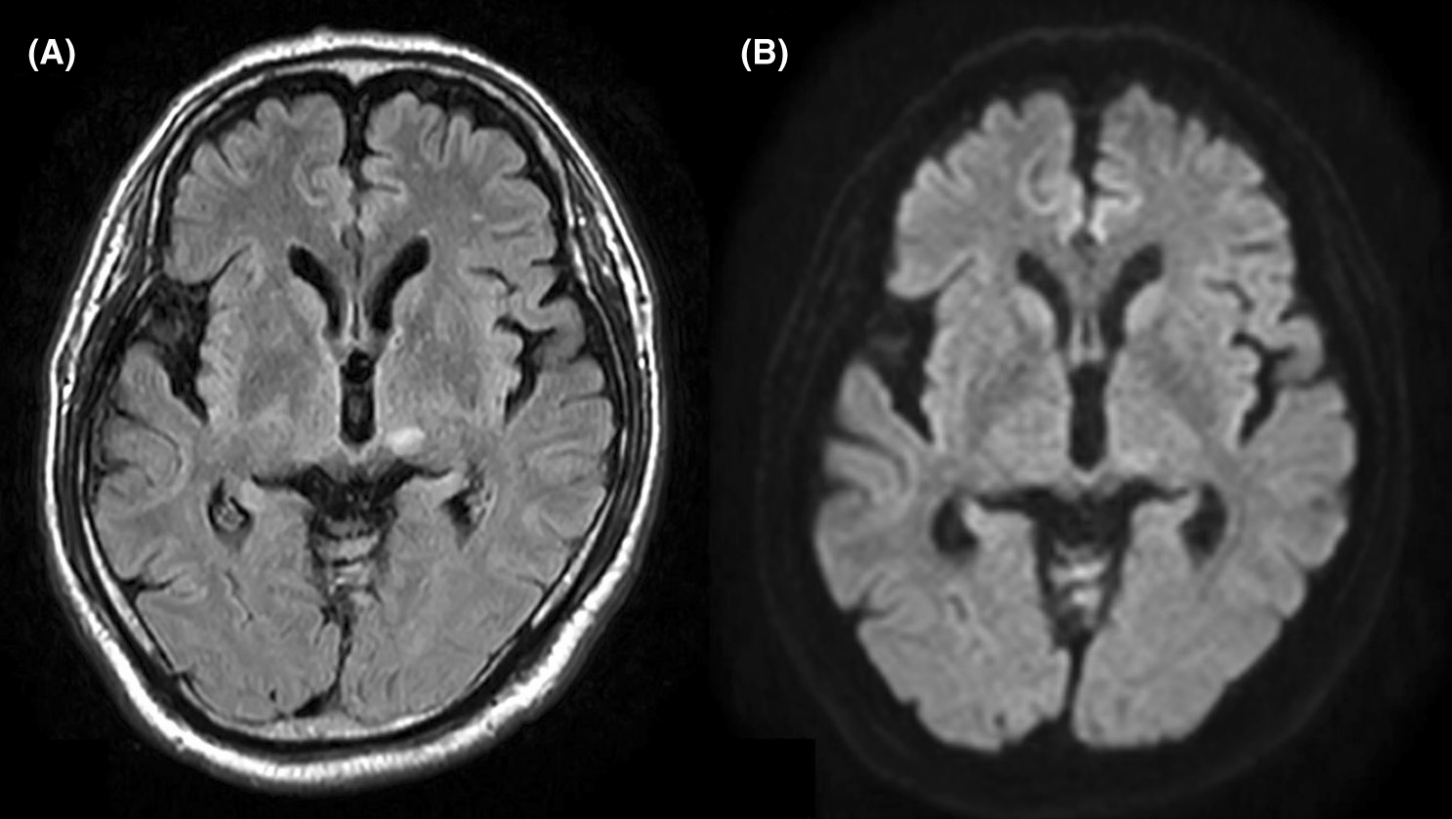
Brain MRI. T2W (A) and FLAIR (B) images show high signal intensity in the left cerebral crus. FLAIR, fluid attenuated inversion recovery.

The patient had a 20 years history of smoking along with dyslipidemia and hyperuricemia, managed with anti‐dyslipidemic and anti‐hyperuricemic medications. Laboratory investigations revealed 0.853 ng/mL of troponin T level and 892 pg/mL of NT‐proBNP level. Electrocardiography showed a regular sinus rhythm with abnormal Q waves and negative T waves in the inferior leads, and ST segment depression in leads V4–6. Also, mild hypokinesis was observed in the inferior area with a left ventricular ejection fraction of 59.6%. Coronary angiography (CAG) revealed total thrombotic occlusion of the ostial right coronary artery (RCA) and no organic stenosis in the left coronary artery (Figure 1). Urgent PCI was performed. The patient was administered with aspirin (200 mg), prasugrel (20 mg), and heparin (9000 international units). An 8‐French Judkins Right catheter (Hyperion JR 3.5, ASAHI INTECC Co., Ltd.) was inserted from the femoral artery and engaged in the RCA, and a 0.014 inch guidewire (ULTIMATE bros 3, ASAHI INTECC Co., Ltd.) was passed into the thrombotic lesion with a microcatheter. Although balloon dilatation (2.5 mm) was performed in the ostium of the RCA (Figure 2A), reperfusion could not be achieved (Figure 2B). Subsequently, aspiration thrombectomy was attempted several times using an aspiration catheter (Rebirth Pro2; NIPRO Co). However, the subsequent CAG showed that a high thrombus burden remained in the RCA (Video S1). After CAG, the patient suddenly developed a headache, dysarthria, and paralysis of the right upper and lower limbs. We suspected acute cerebral infarction, and the neuro‐interventionalist immediately performed cerebral angiography, which showed complete occlusion of the right posterior cerebral artery (Figure 3A). Endovascular thrombectomy was performed by a direct aspiration first‐pass technique using the AXS Catalyst 6 catheter (Stryker Japan K.K.). The tip of the AXS Catalyst 6 catheter reached the occluded site, and the thrombus was aspirated from the tip. The right posterior cerebral artery was successfully reperfused (Figure 3B). No further PCI procedures were performed, and the final thrombolysis in myocardial infarction flow was of grade 2. An intra‐aortic balloon pump (IABP) was added to improve coronary flow and was removed 3 days later. Follow‐up coronary computed tomography angiography revealed a residual thrombus in the RCA after 1 week (Figure 4). A brain MRI showed a high signal in the left cerebral crus on T2‐weighted image (T2WI, Figure 5A)/fluid attenuated inversion recovery (FLAIR, Figure 5B) after 10 days. The infarct size was very small due to emergency endovascular thrombectomy. The patient continued with rehabilitation and was discharged with slight right upper limb paralysis on day 19. He did not complain of chest discomfort or dyspnea upon discharge.

## 3 DISCUSSION

Here, we report a case of embolic stroke during PCI treated with coronary thrombus aspiration. In this case, several balloon dilatations and incomplete aspiration thrombectomy migrated thrombus in the RCA more proximally and the thrombus was subsequently released into the aorta by the backflow of the contrast injection (Video S1).

Major randomized trials and meta‐analyses have concluded that thrombus aspiration is not associated with a reduction in mortality in myocardial infarction.^1^, ^2^, ^3^, ^4^ In a trial of routine aspiration thrombectomy with PCI versus PCI alone in patients with STEMI (TOTAL), manual aspiration thrombectomy was associated with stroke (hazard ratio, 2.08; *p* = 0.002).^2^ Therefore, current guidelines do not recommend routine thrombus aspiration.^5^, ^6^ A previous study elucidated the mechanism of aspiration thrombectomy‐associated stroke involved an incompletely aspirated thrombus dislodging inside the guide catheter and subsequently being injected through the guide catheter into the systemic circulation.^7^ Therefore, it is necessary to open the connector of the guide catheter and drain the thrombus out of the body in order to prevent its release into the systemic circulation.

On the other hand, we captured the moment that the floating thrombus in the RCA is ejected by the backflow of contrast injection, which is an extremely rare mechanism of aspiration thrombectomy‐associated stroke. Because the thrombus is located in the RCA rather than in the guide catheter, it is impossible to drain out of the body as described above. In general, when aspiration thrombectomy is performed, it is important to engage the guide catheter in the ostium of the coronary artery deeply and to use a larger catheter so that the coronary thrombus does not pass through the gap between the guide catheter and the coronary artery. However, when the guide catheter is large, the contrast volume and pressure increase, which can cause displacement of coronary thrombus. Thus, avoiding contrast agent may prevent the aspiration thrombectomy‐associated stroke, but detecting the presence of a floating thrombus require contrast.

Thus, it is very difficult to prevent the rare complication as shown in this case. As the preventive method, intravascular ultrasound (IVUS), which does not require contrast injection, enable to identify the volume and features of the thrombus. Thus, if the IVUS passed the occluded lesion, the evaluation by IVUS can be useful to prevent the embolic complication. Next, a slower and more gentle injection would be also recommended to decrease the contrast pressure. Third, if the patient does not require urgent PCI, since anticoagulation for a few days reduce the thrombus size, it may be considered.

## 4 CONCLUSION

We presented a rare mechanism of stroke complicating coronary thrombus aspiration during PCI. This case suggests that increased thrombus mobility due to thrombus aspiration may be a risk factor for stroke.

## AUTHOR CONTRIBUTIONS

**Reiko Shiomura:** Project administration; writing – review and editing. **Hideki Miyachi:** Supervision. **Takeshi Yamamoto:** Supervision. **Hitoshi Takano:** Supervision.

## FUNDING INFORMATION

No funding was received for this study.

## CONFLICT OF INTEREST

The authors declare no conflicts of interest.

## CONSENT

Written informed consent was obtained from the patient to publish this report in accordance with the journal's patient consent policy.

## Supporting information


Video S1

## Data Availability

The data that support the findings of this study are openly available.
